# Quantitative ^1^H-NMR Spectroscopy for Profiling Primary Metabolites in Mulberry Leaves

**DOI:** 10.3390/molecules23030554

**Published:** 2018-03-02

**Authors:** Qianqian Liang, Qiuying Wang, Yuan Wang, Ya-nan Wang, Jia Hao, Miaomiao Jiang

**Affiliations:** 1 Tianjin State Key Laboratory of Modern Chinese Medicine, Tianjin University of Traditional Chinese Medicine, Tianjin 300193, China; qianqianliang0501@gmail.com (Q.L.); wangqiuying16@gmail.com (Q.W.); haojiasam@tjutcm.edu.cn (J.H.); 2 Guangdong Province Key Laboratory of Pharmacodynamic Constituents of TCM and New Drugs Research, Guangzhou 510632, China; 3 Tianjin Zhongxin Innova Laboratories, Tianjin 300457, China; wangyuan@zx-innova.com; 4 Institute of Materia Medica, Chinese Academy of Medical Sciences & Peking Union Medical College, Beijing 100730, China; wangyanan@imm.ac.cn

**Keywords:** quantitative ^1^H-NMR, *Morus alba*, amino acids, saccharides, organic acids

## Abstract

The primary metabolites in aqueous extract of mulberry (*Morus alba* L.) leaves were characterized by using proton nuclear magnetic resonance (^1^H-NMR) spectroscopy. With the convenience of resonance assignment, GABA together with the other 10 primary metabolites was simultaneously identified and quantified in one ^1^H-NMR spectrum. In this study, external calibration curves for metabolites were employed to calculate the concentrations of interests. The proposed quantitative approach was demonstrated with good linearity (*r^2^* ranged in the interval of 0.9965–0.9999), precision, repeatability, stability (RSD values in the ranges of 0.35–4.89%, 0.77–7.13% and 0.28–2.33%, respectively) and accuracy (recovery rates from 89.2% to 118.5%). The established ^1^H-NMR method was then successfully applied to quantify 11 primary metabolites in mulberry leaves from different geographical regions within a rapid analysis time and a simple sample preparation procedure.

## 1. Introduction

Mulberry leaves are the main food of silkworm, with a long history of industrial use for more than 5000 years [1], and they are also used as functional foods for humans concerning their nutritive and medicinal values. For instance, mulberry tea has been developed to be a popular health food loved by consumers on account of its effects on modulating dyslipidemia, preventing diabetes, and maintaining health [2,3]. The extracts of mulberry leaves are reported to display a wide range of significant biopharmaceutical activities, including antidiabetic, antibacterial, anticancer, cardiovascular, hypolipidemic, antioxidant, antiatherogenic, and anti-inflammatory effects [4]. Phytochemical studies reveal that *γ*-aminobutyric acid (GABA) is one of the principal components isolated from the extracts of mulberry leaves [5]. GABA is well known for its essential role in the nervous system and brain development [6], and may be associated with several of the physiological activities of mulberry leaves, such as antioxidant [7], antihyperglycaemic [8], antihypertensive [9], and anti-inflammatory properties [10]. Like many other aliphatic amino acids, GABA lacks a suitable chromophore for direct ultraviolet detection, and hence, sample derivatization is required before injection to liquid chromatography [11]. However, the pre-column derivatization process is time-consuming, can add impurities into samples, and makes the analysis more complex.

As a result of the improvements in instrumentation and technology, proton nuclear magnetic resonance (^1^H-NMR) has been exploited as a universal and quantitative tool applied in analysis of complex natural samples [12]. An unbiased view in the composition of a given mixture can be offered by ^1^H-NMR spectroscopy with simultaneous access to both qualitative and quantitative information [13]. Quantitative ^1^H-NMR measurement has been proved robust [14] with its error reported to be less than 2.0%, which is an acceptable limit for precise, accurate quantification [15]. In addition, ^1^H-NMR methods provide easy sample preparation, non-destructive analysis, and relatively short analysis times. Compared to mass spectrometry, ^1^H-NMR has from a relatively low detection sensitivity, which can be partly ameliorated by the use of high-field magnets and CryoProbe technologies [12]. With its high capacity to characterize amino acids, organic acids, and carbohydrates without obvious UV chromophores, ^1^H-NMR has become a common quantification tool in many applications for profiling and determining primary metabolites in foods [16,17], plants or herbal remedies [18,19], and biofluid samples [20,21,22]. To the best of our knowledge, the use of a ^1^H-NMR method for chemical profiling of mulberry leaves has not been explored in the literature. In this paper, a rapid, reliable and facile approach based on ^1^H-NMR spectroscopy was developed to identify and quantify GABA together with other primary metabolites in aqueous extracts of mulberry leaves, as well as to provide an alternative method for quality control and evaluation of mulberry leaves in medicinal prescriptions, health care products and functional foods.

## 2. Results and Discussion

### 2.1. Proton Signal Assignments and Chemical Identification

**Figure 1:**
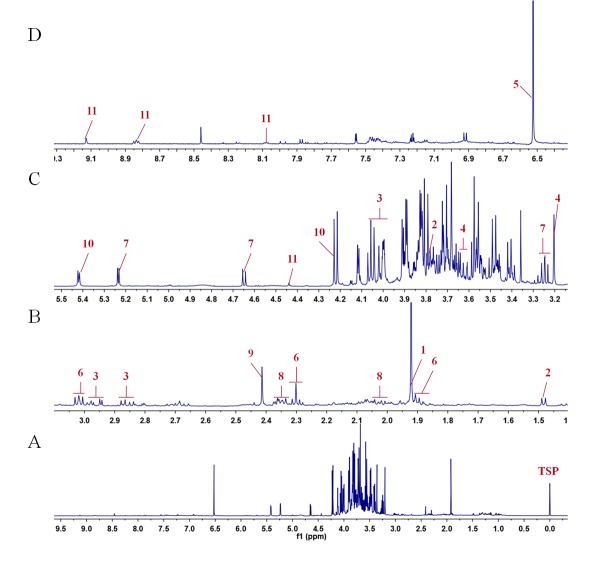
Typical ^1^H-NMR spectra of mulberry leaf extracts. (**A**) Full ^1^H-NMR spectrum from *δ* 0.0 to *δ* 9.5 (TSP-*d*_4_ as an internal standard with chemical shift at *δ* 0.0); (**B**) Enlarged spectrum from *δ* 1.0 to *δ* 3.1; (**C**) Enlarged spectrum from *δ* 3.2 to *δ* 5.5; (**D**) Enlarged spectrum from *δ* 6.4 to *δ* 9.3. Peaks of 11 metabolites: 1, acetic acid; 2, alanine; 3, asparagine; 4, choline; 5, fumaric acid; 6, GABA; 7, glucose; 8, proline; 9, succinic acid; 10, sucrose; 11, trigonelline.

**Figure 2:**
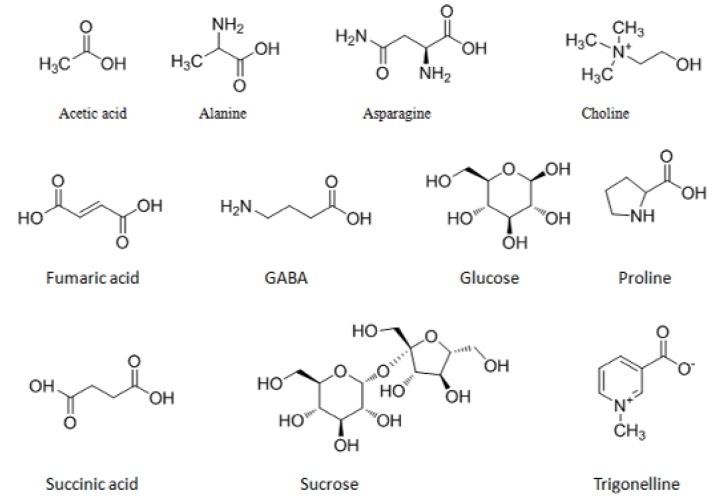
Chemical structures of 11 metabolites from mulberry leaf extracts.

Representative ^1^H-NMR spectra of the aqueous extract of standard material (SM) are shown in Figure 1. The resonance signals were assigned to 11 metabolites according to the published results and further confirmed by a series of 2D NMR spectra. The dominant primary metabolites present in the mulberry leaf extracts included four amino acids (alanine, proline, asparagine and GABA), three organic acids (acetic acid, succinic acid and fumaric acid), two carbohydrates (glucose and sucrose), and two alkaloids (choline and trigonelline). Their chemical structures are shown in Figure 2 and detailed signal assignments are listed in Table 1.

### 2.2. Quantitative ^1^H-NMR Analysis and Method Validation

One inherent advantage of ^1^H-NMR is that the concentrations of substances are directly proportional to the signal areas without any need for response factor correction [12,13]. Routinely, the repetition time should be five times the longest longitudinal relaxation time of resonance T_1_ to measure 99% of the equilibrium magnetization [23].

A long NMR measurement time is required to completely relax all analyzed protons. To reduce the total time spent on multiple scans, an inadequate time of relaxation delay (D_1_ = 4 s) was set to acquire spectra in our work. We then employed external calibration for each metabolite to calculate their contents in extracts to avoid a potential error source of every step of sample treatment and testing [24]. The ^1^H-NMR method was subsequently validated with respect to linearity, precision, repeatability, stability and accuracy (Table 2 and Table 3): 

*Linearity*. Six solutions of 11 metabolites in different concentrations were prepared and analyzed in triplicate. The calibration curve was constructed by plotting the ratio between the peak areas of metabolite and internal standard (x) versus the given metabolite concentration (y). The linear regression equation and correlation coefficient of each metabolite were shown in Table 2. The correlation coefficients of 11 metabolites were in the range of 0.9965–0.9999, indicating good linearity of the established method. In addition, the limit of detection (LOD = 3.3*σ*/*S*) and limit of quantification (LOQ = 10*σ*/*S*) were calculated by the standard deviation of y-intercept of the regression line (σ) and the slope of the calibration curve (S) [25]. The values of LOD and LOQ for 11 metabolites were in the range of 0.001–0.075 mM and 0.002–0.248 mM, respectively.

*Precision.* The intraday precision was determined by analyzing six replicates of the same sample in one day. The relative standard deviation (RSD) values of intraday precision for the contents of 11 metabolites ranged from 0.35% to 4.89%. 

*Stability.* The stability was evaluated by analyzing one sample for 12 h at an interval of every 2 h. The RSD values for the contents of eleven metabolites were in the range of 0.24–2.33%, which showed the analytes were stable during the tested period at ambient temperature. 

*Repeatability.* The repeatability was assessed by analyzing six samples prepared from the same batch of SM. The RSD values of the contents of 11 metabolites ranged from 0.77% to 7.13%, indicating high repeatability. 

*Accuracy.* The accuracy of the NMR method was determined by conventional recovery tests. Six replicates of mixed standard solutions were spiked into 11 known amounts of samples with the same concentration that had previously been analyzed. The spiked samples were then quantified by the established ^1^H-NMR methods. The recovery rate was calculated by 100% × (found amount—original amount)/spiked amount. The results revealed that the average recoveries ranged from 89.2% to 118.5% with RSD values of less than 7.01% for all the 11 metabolites. 

**Figure 3:**
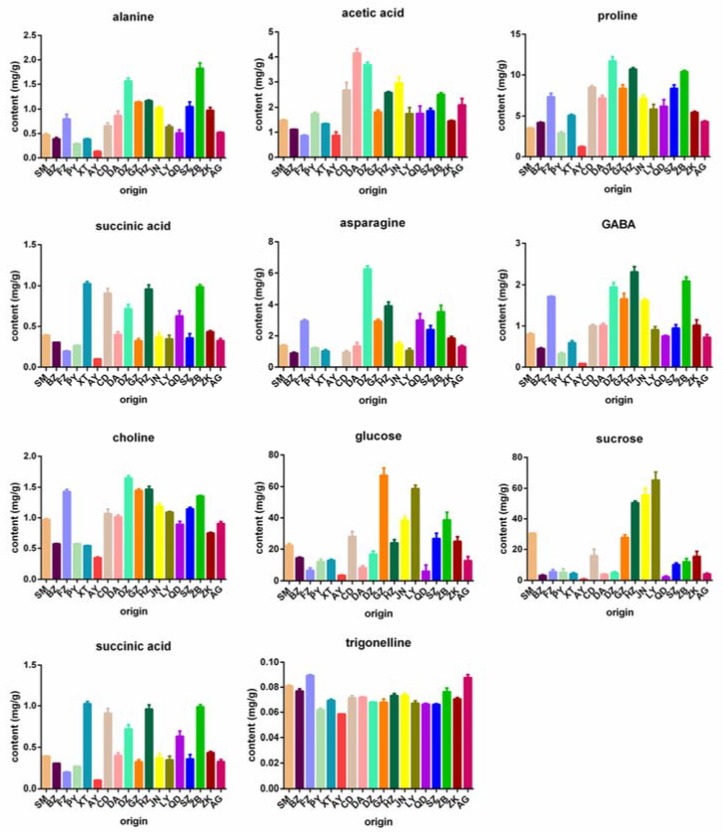
The contents of 11 metabolites in mulberry leaves extracts from 17 origins.

Using the developed ^1^H-NMR method, 11 metabolites were determined simultaneously in 54 samples of mulberry leaves from 18 origins in China (Table S1 and S2), including standard material (SM) obtained from the National Institute for Food and Drug Control (Beijing, China), Pingyao (PY), Anyang (AY), Zhoukou (ZK), Suzhou (SZ), Bozhou (BZ), Guangzhou (GZ), Jinan (JN), Qingdao (QD), Linyi (LY), Heze (HZ), Zibo (ZB), Dezhou (DZ), Anguo (AG), Xingtai (XT), Chengde (CD), Daan (DA), and Fuzhou (FZ). As shown in Figure 3, all 11 metabolites were detected in the samples, except for asparagine that was absent in AY samples. Alanine in BZ, PY, XT and AY samples showed lower levels (0.13–0.39 mg/g) than those of SM (0.48 mg/g) and the other original samples. Acetic acid levels in BZ, FZ, XT, ZK and AY samples ranged from 0.87 mg/g to 1.46 mg/g, less than that of SM samples (1.48 mg/g), while acetic acid in the other origins were in range of 1.74–4.15 mg/g. Proline levels ranged from 3.48 mg/g to 11.71 mg/g, and only those of PY and AY were lower than the standard level (3.53 mg/g). Succinic acid in XT, CD, DZ, HZ, QD and ZB samples showed significantly higher levels (0.63–1.03 mg/g) than that of SM (0.39 mg/g), while BZ, FZ, PY and AY showed relatively low levels. A variation of asparagine from 0 mg/g to 1.30 mg/g was observed in AG, BZ, AY, PY, XT, CD and LY samples, which was much lower than those of SM and the other original samples. GABA in BZ, PY, XT, AY, QD and AG samples displayed lower levels (0.09–0.76 mg/g) than that of SM samples (0.80 mg/g), while GABA in the other origins ranged from 0.91–2.30 mg/g. Choline levels from most origins were higher than that of SM (0.97 mg/g) except the origins of BZ, PY, XT, AY, ZK and AG. Glucose and sucrose varied in wide ranges of 3.41–64.23 mg/g and 0.72–65.41 mg/g, respectively. Fumaric acid was detected in lower levels from FZ, AY, DA and SZ origins (0.05–0.19 mg/g) than the standard level (5.43 mg/g). Trigonelline varied in a range of 0.06–0.09 mg/g, which paralleled to that of SM (0.08 mg/g). These data indicated that the contents of 10 primary metabolites changed obviously with different geographical origins.

### 2.3. The Metabolic Variations of Mulberry Leaves from Different Origins

**Figure 4:**
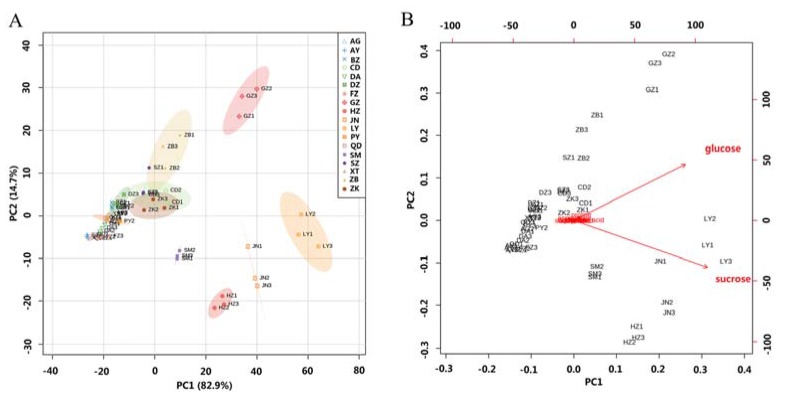
Scores plot (**A**) and biplot (**B**) of PCA model established with the quantitative NMR data.

Principal component analysis (PCA) was carried out with the quantitative NMR data to simulate the metabolic trends of all the samples obtained from different regions. Scores plot (Figure 4A) showed that samples from the same region clustered well, whereas the ones from LY, GZ, JN, HZ and SM origins were separable. On the direction of PC2, samples from HZ, LY and JN were close to the standard samples, which may be caused by similar geographic and climate conditions of the three regions. Biplot of PCA (Figure 4B) further revealed that glucose and sucrose major contributed to the classification, which was consistent with the content determination results. They are important sources of energy for cellular respiration in plants.

**Figure 5:**
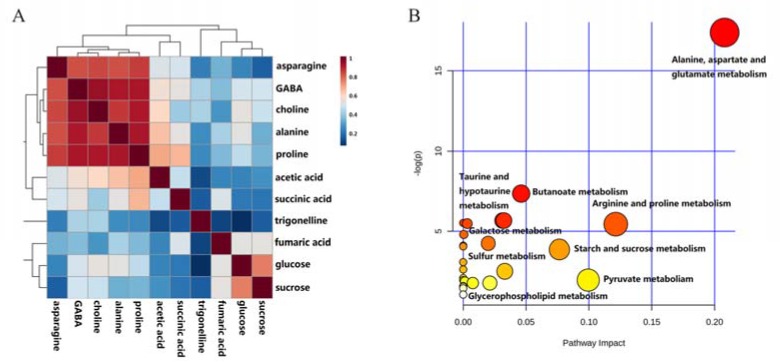

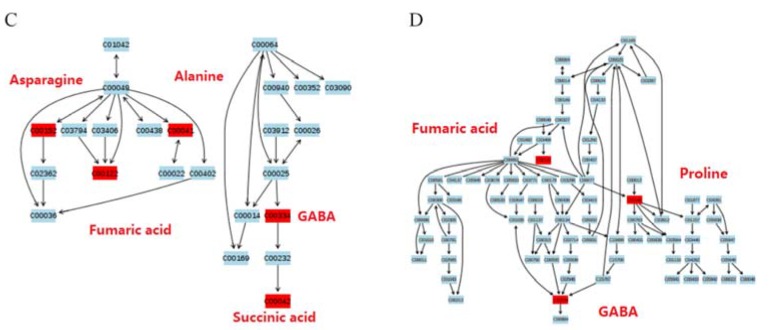
(**A**) Correlation analysis of 11 metabolites from mulberry leaves extracts; (**B**) Overview of pathway analysis with 11 metabolites from mulberry leaves extracts; (**C**) Alanine, aspartate and glutamate metabolism; (**D**) Arginine and proline metabolism.

Correlation analysis showed an obviously positive relationship among asparagine, GABA, choline, alanine, and proline (Figure 5A), indicating they had a similar variation tendency. It was further confirmed by the results of pathway enrichment analysis that asparagine, GABA, alanine and proline were subjected to two pathways with high impact values (Figure 5B), including alanine, aspartate and glutamate metabolism (Figure 5C) as well as arginine and proline metabolism (Figure 5D). GABA as an intermediate in the two pathways, and our results suggested that inhibiting transamination of GABA might increase its yield in this plant.

## 3. Materials and Methods 

### 3.1. Materials and Reagents

Proline (≥99%), choline chloride (≥98%) and sodium acetate (≥98%) were purchased from the National Institute for Food and Drug Control (Beijing, China). D-(+)-Glucose (99.9%) was purchased from Supelco (Bellefonte, PA, USA). Sucrose (≥99.5%) was purchased from Shanghai Aladdin Biological Technology Co., Ltd. (Shanghai, China). L-Asparagine (≥98%), succinic acid (analytical standard), trigonelline hydrochloride (analytical standard), L-alanine (≥98%) and sodium hydrogen fumarate (≥99%) were purchased from Sigma (St. Louis, MO, USA). γ-Aminobutyric acid (GABA, ≥99%) was purchased from Shanghai Yuanye Biological Technology Co., Ltd. (Shanghai, China). Deuterium oxide (D_2_O, 99.9 atom % D) and sodium 3-trimethylsilyl [2,2,3,3-*d*_4_] propionate (TSP-*d*_4_, 98 atom % D) were from Cambridge Isotope Laboratories (Cambridge, FL, USA).

The standard material (SM) of mulberry leaves (*Morus alba* L) was obtained from the National Institute for Food and Drug Control (Beijing, China). The other samples of mulberry leaves were collected from 17 cities in China (Table S3), including Pingyao (PY), Anyang (AY), Zhoukou (ZK), Suzhou (SZ), Bozhou (BZ), Guangzhou (GZ), Jinan (JN), Qingdao (QD), Linyi (LY), Heze (HZ), Zibo (ZB), Dezhou (DZ), Anguo (AG), Xingtai (XT), Chengde (CD), Daan (DA), and Fuzhou (FZ).

### 3.2. Sample Preparation

The mulberry leaves were dried at 80 °C for 4 h and ground to a fine powder. Six hundred microliters of D_2_O containing 0.1177 mM TSP-*d_4_* were added into 24 mg of leaf powder as extraction solvent. D_2_O was used for the internal lock signal and TSP-*d_4_* as the internal standard with a chemical shift of *δ* 0.0. The extracts were impregnated for 1 h, and sonicated for 15 min, followed by centrifugation for 10 min (14,000 rpm) at room temperature. The supernatants were transferred into 5 mm NMR tubes (Vineland, NJ, USA) for further analysis.

### 3.3. NMR Spectroscopy

All the NMR spectra were acquired at 298 K on a 600 MHz Bruker AVIII HD spectrometer (Bruker BioSpin, Rheinstetten, Germany) equipped with a 5 mm BBO H&F cryogenic probe. Automatic shimming and adjusting of 90° pulse length were performed for each sample. Standard Carr-Purcell-Meiboom-Gill (CPMG) pulse sequence with water presaturation was used to record the ^1^H-NMR spectra for filtering the broad signals from the molecules (such as proteins) with short T_2_ relaxation times. A total of 64 scans were collected into 64 K data points over a spectral width of 12,019.2 Hz with a relaxation delay of 4 s and an acquisition time of 3.0671 s. An exponential line-broadening of 0.3 Hz was applied to the free induction decay prior to Fourier transformation. Additional ^1^H, ^1^H-correlation spectroscopy (COSY), ^1^H-^1^H total correlation spectroscopy (TOCSY) and ^1^H, ^13^C-heteronuclear single quantum correlation (HSQC) spectra were recorded on the selected samples for the purpose of resonance assignment.

### 3.4. Quantification of the Metabolites

The original NMR data were processed by MestReNova 6.1.0 (Mestrelab Research S. L., Santiago de Compostela, Spain) with automatic correction of phase and baseline. Quantitative signals of 11 metabolites were selected to integrate manually. Despite the integral areas directly proportional to the number of contributing protons, the accuracy of measurements can also be influenced by various experimental parameters and conditions, such as pulse sequence, relaxation delay, and the purity of the internal standard. We thus employed external calibration curve to calculate the content of each metabolite. Six different concentration solutions of acetic acid, alanine, asparagine, choline, fumaric acid, GABA, glucose, proline, succinic acid, sucrose and trigonelline in the ranges of 0.1848–5.1925 mM, 0.0288–0.9204 mM, 0.1650–5.2816 mM, 0.0895–2.8656 mM, 0.4298–13.7509 mM, 0.0640–2.0413 mM, 1.2417–39.7322 mM, 0.1928–6.1634 mM, 0.0350–1.1179 mM, 0.3111–9.9562 mM and 0.0265–0.8450 mM were employed to establish the linear regression equations according to the ratio between the peak areas of metabolites and internal standard (x) versus the concentration of given metabolites (y).

### 3.5. Statistical Analysis

All data were expressed as mean values ± standard deviation and performed using GraphPad Prism 6.04 software (GraphPad Software, Inc., San Diego, CA, USA). Principal component analysis (PCA) analysis, correlation analysis and pathway analysis based on Kyoto Encyclopedia of Genes and Genomes (KEGG) database were carried out with MetaboAnalyst 3.0 (http://www.metaboanalyst.ca/). 

## 4. Conclusions

A quantitative ^1^H-NMR method was established to identify and determine 11 primary metabolites in the leaves of *Morus alba* L. External calibration curves were employed for quantitative measurements, and subsequent validation results showed the proposed approach having good linearity, precision, repeatability and accuracy. Compared to conventional HPLC-UV spectroscopy, the developed ^1^H-NMR approach enables a robust and non-destructive determination of primary metabolites in the extracts of mulberry leaves without any derivatization or separation procedures. Multivariate statistical analyses revealed that GABA, asparagine, choline, alanine, and proline were probably varied with geographical regions, while trigonelline had no obvious in different origins.

## Figures and Tables

**Table 1:** Proton NMR spectroscopic data for 11 metabolites in D_2_O.

No.	Metabolites	δ H (Multiplicity ^a^)	Assignment
1	Acetic acid	1.91 (s)	CH_3_
2	Alanine	3.78 (q), 1.48 (d)	*α*-CH, *β*-CH_3_
3	Asparagine	2.86 (dd), 2.96 (dd), 4.02 (dd)	β-CH_2_, α-CH
4	Choline	4.11 (m), 3.52 (dd), 3.21 (s)	2-CH_2_, 3-CH_2_, 4-N(CH_3_)_3_
5	Fumaric acid	6.50 (s)	CH
6	GABA	3.00 (t), 2.30 (t), 1.90 (m)	*γ*-CH_2_, *α-*CH_2_, *β-*CH_2_
7	Glucose	5.24 (d), 4.65 (d), 3.89 (m), 3.83 (m), 3.75 (m), 3.69 (m), 3.52 (m), 3.48 (m), 3.40 (m), 3.25 (dd)	2-CH, 11-CH, 6-CH, 4-CH, 3-CH, 5-CH
8	Proline	4.11 (m), 3.41 (m), 3.28 (m), 2.35 (m), 2.04 (m)	*α-*CH, *δ-*CH_2_, *β-*CH_2_, *γ*-CH_2_
9	Succinic acid	2.41 (s)	CH_2_
10	Sucrose	5.40 (d), 4.20 (d), 4.04 (t), 3.80 (m), 3.55 (m), 3.46 (t)	7-CH, 3-CH, 4-CH, 17-CH, 19-CH, 12-CH, 10-CH
11	Trigonelline	9.13 (s), 8.84 (t), 8.09 (t), 4.44 (s)	1-CH, 3-CH, 5-CH, 4-CH, 9-CH_3_

^a^ Abbreviations, s = singlet, d = doublet, dd = doublet-doublets, m = multiplet, t = triplet.

**Table 2:** The linear regression equations, LOD and LOQ for 11 metabolites (*n* = 3).

No.	Metabolites	Regression Equation	Correlation Coefficients (*r^2^)*	LOD (mM)	LOQ (mM)
1	Acetic acid	y = 0.3087x + 0.1805	0.9993	0.040	0.132
2	Alanine	y = 0.5976x + 0.0173	0.9998	0.012	0.038
3	Asparagine	y = 1.2754x + 0.0969	0.9996	0.025	0.083
4	Choline	y = 0.0849x + 0.0916	0.9965	0.001	0.002
5	Fumaric acid	y = 0.5585x + 0.0085	0.9999	0.003	0.009
6	GABA	y = 0.7050x + 0.0319	0.9996	0.016	0.053
7	Glucose	y = 2.7552x + 0.5830	0.9995	0.075	0.248
8	Proline	y = 1.4021x + 0.1328	0.9982	0.036	0.121
9	Succinic acid	y = 0.1945x + 0.0316	0.9993	0.004	0.013
10	Sucrose	y = 3.3043x + 0.0558	0.9981	0.047	0.156
11	Trigonelline	y = 0.0819x + 0.0170	0.9993	0.003	0.009

**Table 3:** The methodological investigation results of precision, stability, repeatability and recovery (*n* = 6).

Metabolites	Precision	Stability	Repeatability	Recovery	
	RSD (%)	RSD (%)	RSD (%)	(%)	RSD (%)
Acetic acid	0.35	0.28	1.32	106.1	5.46
Alanine	2.39	0.94	5.15	91.6	7.01
Asparagine	4.89	2.33	2.65	118.5	5.25
Choline	0.69	0.24	1.09	109.8	2.26
Fumaric acid	1.15	0.32	0.77	90.4	4.47
GABA	4.21	1.74	2.48	95.6	2.52
Glucose	0.68	0.75	3.82	116.2	1.53
Proline	0.90	0.79	2.00	90.7	2.83
Succinic acid	0.75	0.46	0.85	89.2	1.70
Sucrose	2.40	1.81	7.13	99.8	4.34
Trigonelline	1.27	0.30	0.80	104.0	3.07

## Supplementary Materials

Supplementary Materials are available on line. Table S1 and S2. The contents of 11 metabolites in the extracts of mulberry leaves from 18 origins. Table S3. The geographical information of 18 different regions.

## Abbreviations

^1^H-NMR	Proton nuclear magnetic resonance
GABA	*γ*-Aminobutyric acid
TSP-*d_4_*	Sodium 3-trimethylsilyl [2,2,3,3-*d*_4_] propionate
D_2_O	Deuterium oxide
CPMG	Carr-Purcell-Meiboom-Gill
COSY	^1^H, ^1^H-correlation spectroscopy
TOCSY	^1^H-^1^H total correlation spectroscopy
HSQC	^1^H, ^13^C-heteronuclear single quantum correlation
PCA	Principal component analysis
SM	Standard material
PY	Pingyao
AY	Anyang
ZK	Zhoukou
SZ	Suzhou
BZ	Bozhou
GZ	Guangzhou
JN	Jinan
QD	Qingdao
LY	Linyi
HZ	Heze
ZB	Zibo
DZ	Dezhou
AG	Anguo
XT	Xingtai
CD	Chengde
DA	Daan
FZ	Fuzhou
