# Supplementary material for: Quantitative 1H-NMR Spectroscopy for Profiling Primary Metabolites in Mulberry Leaves
Source: Molecules. 2018 Mar 2;23(3):554. doi: 10.3390/molecules23030554 (PMC6017862; doi:10.3390/molecules23030554)
Supplement: Supplementary file 1 [file molecules-23-00554-s001.doc]

**Quantitative ^1^H-NMR Spectroscopy for Profiling** **Primary Metabolites in Mulberry Leaves**

Qianqian Liang ^1,2^, Qiuying Wang ^1^, Yuan Wang ^3^, Ya-nan Wang ^4^, Jia Hao ^1^ and Miaomiao Jiang ^1^,*

1. Tianjin State Key Laboratory of Modern Chinese Medicine, Tianjin University of Traditional Chinese Medicine, Tianjin 300193, China; qianqianliang0501@gmail.com (Q.L.); wangqiuying16@gmail.com (Q.W.); haojiasam@tjutcm.edu.cn (J.H.)
2. Guangdong Province Key Laboratory of Pharmacodynamic Constituents of TCM and New Drugs Research, Guangzhou 510632, China
3. Tianjin Zhongxin Innova Laboratories, Tianjin 300457, China; wangyuan@zx-innova.com
4. Institute of Materia Medica, Chinese Academy of Medical Sciences & Peking Union Medical College, Beijing 100730, China; wangyanan@imm.ac.cn

***** Correspondence: miaomiaojiang@tjutcm.edu.cn; Tel.: +86-022-5959-6163

**Table S1.** The content of 11 metabolites in samples from nine origins (n=3).

| **Metabolites** | **Mean ± SD (mg/g)** | | | | | | | | |
| --- | --- | --- | --- | --- | --- | --- | --- | --- | --- |
|  | **AG** | **BZ** | **FZ** | **PY** | **XT** | **AY** | **CD** | **DA** | **DZ** |
| Alanine | 0.52±0.01 | 0.39±0.03 | 0.80±0.09 | 0.29±0.01 | 0.38±0.01 | 0.13±0.01 | 0.66±0.06 | 0.87±0.09 | 1.57±0.06 |
| Acetic acid | 2.10±0.26 | 1.12±0.01 | 0.87±0.03 | 1.75±0.06 | 1.34±0.02 | 0.87±0.14 | 2.67±0.30 | 4.15±0.18 | 3.69±0.10 |
| Proline | 4.32±0.10 | 4.20±0.06 | 7.37±0.40 | 2.90±0.19 | 5.08±0.14 | 1.21±0.07 | 8.47±0.22 | 7.21±0.32 | 11.71±0.53 |
| Succinic acid | 0.34±0.11 | 0.31±0.01 | 0.20±0.01 | 0.27±0.01 | 1.03±0.03 | 0.10±0.01 | 0.91±0.06 | 0.40±0.03 | 0.72±0.05 |
| Asparagine | 1.30±0.08 | 0.90±0.05 | 2.95±0.09 | 1.21±0.04 | 1.03±0.09 | - | 0.94±0.11 | 1.34±0.22 | 6.26±0.21 |
| GABA | 0.73±0.06 | 0.45±0.02 | 1.72±0.01 | 0.33±0.03 | 0.59±0.05 | 0.09±0.01 | 1.01±0.03 | 1.01±0.05 | 1.95±0.11 |
| Choline | 0.91±0.03 | 0.57±0.01 | 1.43±0.04 | 0.57±0.01 | 0.54±0.01 | 0.35±0.01 | 1.07±0.07 | 1.01±0.03 | 1.65±0.04 |
| Glucose | 12.91±2.60 | 14.71±0.47 | 6.69±1.59 | 12.24±1.60 | 13.10±0.60 | 3.41±0.27 | 28.06±3.25 | 8.37±1.28 | 16.71±2.05 |
| Sucrose | 4.25±0.50 | 3.22±0.46 | 5.57±1.56 | 5.21±2.47 | 4.35±0.72 | 0.72±0.37 | 16.07±4.18 | 3.95±0.25 | 4.97±0.83 |
| Fumaric acid | 1.75±0.61 | 4.80±0.07 | 0.07±0.01 | 2.99±0.06 | 3.83±0.10 | 0.05±0.01 | 5.75±0.15 | 0.07±0.01 | 7.15±0.17 |
| Trigonelline | 0.09±0.01 | 0.08±0.01 | 0.09±0.01 | 0.06±0.01 | 0.07±0.01 | 0.06±0.01 | 0.07±0.01 | 0.07±0.01 | 0.07±0.01 |

**Table S2. The content of 11 metabolites in samples from nine origins (n=3).**

| **Metabolites** | **Mean ± SD (mg/g)** | | | | | | | | |  |
| --- | --- | --- | --- | --- | --- | --- | --- | --- | --- | --- |
|  | **GZ** | **HZ** | **JN** | **LY** | **QD** | **SZ** | **ZB** | **ZK** | **SM** | |
| Alanine | 1.14±0.01 | 1.17±0.02 | 1.02±0.03 | 0.63±0.04 | 0.51±0.06 | 1.05±0.10 | 1.83±0.11 | 0.95±0.02 | 0.48±0.02 | |
| Acetic acid | 1.81±0.09 | 2.57±0.04 | 2.96±0.25 | 1.74±0.24 | 1.75±0.30 | 1.86±0.10 | 2.52±0.05 | 1.46±0.02 | 1.48±0.03 | |
| Proline | 8.35±0.44 | 10.72±0.17 | 7.18±0.37 | 5.86±0.58 | 6.20±0.83 | 8.19±0.44 | 10.42±0.14 | 5.46±0.12 | 3.53±0.05 | |
| Succinic acid | 0.32±0.02 | 0.96±0.05 | 0.37±0.05 | 0.35±0.05 | 0.63±0.06 | 0.36±0.05 | 0.99±0.03 | 0.443±0.01 | 0.39±0.01 | |
| Asparagine | 2.94±0.11 | 3.92±0.25 | 1.48±0.14 | 1.06±0.14 | 3.01±0.41 | 2.40±0.26 | 3.54±0.42 | 1.85±0.11 | 1.38±0.05 | |
| GABA | 1.66±0.14 | 2.30±0.13 | 1.62±0.05 | 0.91±0.08 | 0.76±0.02 | 0.95±0.08 | 2.10±0.08 | 1.02±0.13 | 0.80±0.02 | |
| Choline | 1.45±0.03 | 1.47±0.05 | 1.19±0.05 | 1.09±0.01 | 0.89±0.05 | 1.15±0.03 | 1.36±0.01 | 0.75±0.01 | 0.97±0.01 | |
| Glucose | 64.23±4.68 | 24.02±2.11 | 38.54±2.46 | 58.67±2.22 | 6.15±3.93 | 26.79±3.46 | 38.67±4.88 | 25.00±2.88 | 22.47±0.07 | |
| Sucrose | 27.86±1.75 | 50.56±1.22 | 55.54±4.61 | 65.41±5.24 | 2.22±0.73 | 10.35±1.17 | 12.21±2.10 | 15.72±3.07 | 30.56±0.24 | |
| Fumaric acid | 2.79±0.74 | 4.69±0.62 | 4.57±0.54 | 8.55±0.60 | 3.57±0.22 | 0.19±0.03 | 6.46±0.13 | 9.70±0.34 | 5.43±0.06 | |
| Trigonelline | 0.07±0.01 | 0.07±0.01 | 0.07±0.01 | 0.07±0.01 | 0.07±0.01 | 0.07±0.01 | 0.08±0.01 | 0.07±0.01 | 0.08±0.01 | |

**Table S3.** The geographical information of the 18 different regions

| **Origin (abbreviation)** | **Province** | **Longitude** | **Latitude** |
| --- | --- | --- | --- |
| **SM** | Beijing | 116:28E | 39:54N |
| **AG** | Hebei | 115.33E | 38.42N |
| **XT** | Hebei | 114.51E | 37.07N |
| **CD** | Hebei | 117:52E | 40:59N |
| **DA** | Jilin | 124.29E | 45.51N |
| **FZ** | Fujian | 119.30E | 26.08N |
| **PY** | Shanxi | 112.18E | 37.19N |
| **GZ** | Guangdong | 113:18E | 23:10N |
| **SZ** | Anhui | 116.97E | 33.65N |
| **BZ** | Anhui | 115.78E | 33.85N |
| **ZB** | Shandong | 118.06E | 36.81N |
| **HZ** | Shandong | 115.48E | 35.23N |
| **JN** | Shandong | 117:02E | 36:40N |
| **LY** | Shandong | 118.36E | 35.11N |
| **QD** | Shandong | 120:19E | 36:04N |
| **DZ** | Shandong | 116.36E | 37.44N |
| **AY** | Henan | 114.39E | 36.10N |
| **ZK** | Henan | 114.70E | 33.63N |
